# Prostate cancer screening in the Middle East and North Africa: a cross-sectional study on current practices

**DOI:** 10.1093/jncics/pkaf019

**Published:** 2025-02-08

**Authors:** Ozlem Aynaci, Yetkin Tuac, Layth Mula-Hussain, Lubna Hammoudeh, Salameh Obeidat, Enas Abu Abeelh, Ahmed H Ibrahim, Sepideh Mohammadipour, Bader Alali, Ahmed Jdaini, Ali Barki, Nesrine Mejri, Zeinab Alhaddad, Nadeem Pervez, Hussain Al Hussain, Mohamad Kadri, Mohamed A Elfagieh, Adda Bounedjar, Moamin Junaid, Ahmed M Badheeb, Ibrahim Abu Ghida, Shalini Moningi, Jonathan E Leeman, Peter F Orio, Paul L Nguyen, Anthony V D’Amico, Mutlay Sayan

**Affiliations:** Karadeniz Technical University, Trabzon 61080, Türkiye; Department of Statistics, Ankara University, Ankara 06100, Türkiye; Faculty of Medicine, Dalhousie University, Halifax, NS B3H 4R2, Canada; College of Medicine, Ninevah University, Mosul, Ninevah 41002, Iraq; Oregon Health and Science University, Portland, OR 97239, United States; Oregon Health and Science University, Portland, OR 97239, United States; Primary Health Care Corporation, Doha, Qatar; Ain Shams University, Cairo 11566, Egypt; Hacettepe University, Ankara 06100, Türkiye; Jaber Al Ahmad Hospital, Kuwait, Kuwait; Mohammed VI University Medical Center, Oujda 60050, Morocco; Mohammed VI University Medical Center, Oujda 60050, Morocco; Abderrahmen Mami Hospital, Ariana 2080, Tunisia; King Hamad University Hospital, Al Sayh, Bahrain; Sheikh Shakhbout Medical City, Abu Dhabi 11001, UAE; King Fahd Medical City, Riyadh 12231, Saudi Arabia; AlBaironi Hospital, Damascus, Syria; National Cancer Institute, Misurata, Libya; University of Blida, Blida 09000, Algeria; University of Ninevah, Mosul 41002, Iraq; Hadhramout University, Mukalla, Yemen; Burjeel Medical City, Abu Dhabi, United Arab Emirates; Emirates Oncology Society, Dubai, United Arab Emirates; Department of Radiation Oncology, Brigham and Women’s Hospital and Dana-Farber Cancer Institute, Harvard Medical School, Boston, MA 02115, United States; Department of Radiation Oncology, Brigham and Women’s Hospital and Dana-Farber Cancer Institute, Harvard Medical School, Boston, MA 02115, United States; Department of Radiation Oncology, Brigham and Women’s Hospital and Dana-Farber Cancer Institute, Harvard Medical School, Boston, MA 02115, United States; Department of Radiation Oncology, Brigham and Women’s Hospital and Dana-Farber Cancer Institute, Harvard Medical School, Boston, MA 02115, United States; Department of Radiation Oncology, Brigham and Women’s Hospital and Dana-Farber Cancer Institute, Harvard Medical School, Boston, MA 02115, United States; Department of Radiation Oncology, Brigham and Women’s Hospital and Dana-Farber Cancer Institute, Harvard Medical School, Boston, MA 02115, United States

## Abstract

**Background:**

Prostate cancer is a substantial health concern in the Middle East and North Africa region, with many cases diagnosed at advanced stages, a high mortality to incidence ratio, and low prostate cancer awareness. This study aimed to evaluate prostate cancer screening practices in the region to inform effective early detection and management strategies.

**Methods:**

A cross-sectional survey was conducted from July 1, 2023, to November 8, 2024, among physicians from 19 countries in the Middle East and North Africa region. The study used a validated questionnaire to assess prostate cancer screening practices, barriers, and educational needs.

**Results:**

The survey had a response rate of 96.8% and 1163 participants. Of these participants, 34.7% routinely performed prostate cancer screenings, with 61.1% using prostate-specific antigen tests. The primary barrier was lack of patient awareness (51.2%). In addition, 65.3% of participants had no formal training. To improve screening rates, participants suggested better patient education (63.5%), increased training for health-care professionals (41.9%), and improved access to screening equipment (38.9%).

**Conclusion:**

This study revealed that prostate cancer screening rates were low, with barriers including a lack of patient awareness and formal training among physicians. Addressing these issues through culturally tailored education programs may improve early detection rates and ultimately reduce the burden of prostate cancer in the Middle East and North Africa region.

## Introduction

Prostate cancer is a major global health concern, yet it remains understudied in the Middle East and North Africa because of a lack of comprehensive cancer registries.^1-3^ Although data are limited, small retrospective studies indicate that a statistically significant number of men in the Middle East and North Africa region are diagnosed with advanced-stage prostate cancer, largely because of symptomatic presentation rather than proactive screening.^4-7^ This point is supported by findings from a retrospective analysis of 1136 prostate patients with cancer across 6 countries in the Middle East and North Africa region, which reported that 78% of the patients were diagnosed only after exhibiting symptoms rather than through routine prostate-specific antigen (PSA) screening.^7^ Compounding this concern is the region’s high mortality to incidence ratio for prostate cancer, which is significantly higher than that of Europe and North America.^8^^,^^9^ In addition, a recent cross-sectional study assessing prostate cancer awareness among men in the Middle East and North Africa region with a 74.9% response rate from 4431 participants highlighted that although 83.8% of the participants had heard of prostate cancer, misconceptions persisted, with 21.8% of respondents mistakenly believing that it affects both sex and only 19.1% being aware of PSA screening.^10^ This disparity is further exacerbated by the lack of comprehensive data on prostate cancer screening practices in this region.

Prostate cancer screening is associated with a decrease in prostate cancer–specific mortality.^11-15^ Although the Prostate, Lung, Colorectal, and Ovarian Cancer Screening Trial initially found no significant reduction in 10-year prostate cancer–specific mortality with screening,^16^^,^^17^ partly because of high contamination rates (nearly 50% of men in the no-screening arm were still receiving PSA tests), the European Randomized Study of Screening for Prostate Cancer trial demonstrated a 20% reduction in prostate cancer–specific mortality with routine PSA screening.^11-13^ A subsequent reanalysis of these trials suggested that PSA screening could reduce prostate cancer–specific mortality by approximately 30% when accounting for contamination and adherence differences.^15^ Consequently, major guidelines now recommend a risk-adapted, shared decision-making approach to screening.^18-21^ Given that nearly 50% of patients in the Middle East and North Africa region present with metastatic prostate cancer^7^ along with a high mortality to incidence ratio^8^^,^^9^ and low prostate cancer awareness,^10^ it is prudent to understand the status of prostate cancer screening in this region. The present study addressed this urgent need by evaluating prostate cancer screening practices across the Middle East and North Africa region, aiming to provide insights that could inform the development of effective early detection and management strategies, ultimately improving prostate cancer outcomes across the region.

## Methods

### Study design and participants

This cross-sectional survey was conducted between July 1, 2023, and November 8, 2024, to investigate the status of prostate cancer screening in the Middle East and North Africa region. The study focused on physicians residing in the following 19 countries: Algeria, Bahrain, Egypt, Iran, Iraq, Israel, Jordan, Kuwait, Lebanon, Libya, Morocco, Oman, Qatar, Saudi Arabia, Syria, Tunisia, Türkiye, the United Arab Emirates, and Yemen. To provide comprehensive representation of the region, to maintain consistency with prior publications,^9^^,^^22^ and in accord with the World Health Organization’s International Agency for Research on Cancer’s Global Cancer Observatory database,^23^ we sought to include participants from the Gaza Strip and West Bank and chose this phrasing for consistency.

### Data-collection tool

A questionnaire was used as the primary data-collection tool for this study (Supplementary material). It was validated by experts in the field, who reviewed the content for relevance, clarity, and appropriateness. To ensure contextual relevance and accuracy, this process was conducted by 5 oncologists who reside and practice within the Middle East and North Africa region. These oncologists were selected based on their specialized expertise in prostate cancer and experience within the regional health-care environment.

The questionnaire comprehensively assessed participants’ screening practices, including the frequency and methods of prostate cancer screening, barriers faced in conducting screenings, and factors that could potentially improve screening rates. In addition, it collected demographic information, such as age, sex, and professional background, and evaluated participants’ knowledge and educational needs regarding prostate cancer screening. This approach ensured a thorough understanding of the current screening landscape, and the challenges health-care professionals encounter in the Middle East and North Africa region.

Recruitment was managed by participating physicians, who primarily targeted colleagues at their facilities. Although the primary targets were general practitioners, urologists, and oncologists, the selection process was conducted at the discretion of the local team leaders, allowing for flexibility to accommodate differences in health-care systems across various countries. The questionnaire was administered to participants through various methods, including verbal administration by telephone and in-person interviews, as well as electronic distribution. Participation in the study was entirely voluntary, and no incentives were offered.

### Sample size determination

The sample size was calculated using OpenEpi software (Emory University). The calculations were based on a 95% confidence interval and a 5% margin of error, assuming a 50% prevalence rate for prostate cancer screening. To account for potential nonresponses, an additional 10% was included in the sample size. This calculation yielded a final sample size of 1202 participants. To ensure that the data-collection process was feasible and practical, an initial pilot study with 20 participants was conducted. During this pilot phase, both the questionnaire and the logistical arrangements were evaluated. Feedback from participants indicated that the questionnaire and data-collection procedures were well received and suitable for implementation in the larger study.

To ensure balanced representation across the study, the goal was to include at least 66 participants from each location. By the end of the study, a total of 1163 participants were successfully recruited, with the number of participants from each location ranging from 22 to 84, ensuring a representative sample without significant outliers.

To prevent duplicate responses, each participant was assigned a unique identifier by local team leaders, ensuring 1 response per person. The data-collection process was closely monitored across verbal, in-person, and electronic methods to prevent multiple entries. Before analysis, the data were thoroughly cleaned to remove any duplicate entries.

### Statistical analysis

Statistical analyses were conducted using IBM SPSS, version 25, software. Because the survey data were categorical, frequencies and percentages were calculated for descriptive statistics. The relationships between these characteristics were assessed using the Pearson χ^2^ test and in cases of small sample sizes—such as the association between the age group of participants and their preferences for performing prostate cancer screening—the Fisher exact test was used. To investigate the factors influencing the participants’ preferences for PSA screening, a logistic regression analysis was performed.

The study was performed in accordance with the Declaration of Helsinki and the principles of Good Clinical Practice. The study protocol was reviewed and approved by the Institutional Review Board of Karadeniz Technical University (Trabzon, Türkiye). Written informed consent for participation was not required for this study in accordance with national legislation and institutional requirements.

## Results

A total of 1163 participants completed the survey, resulting in a response rate of 96.8%. The majority of participants (69%) were male and ranged in age from 30 to 39 years (34.7%); 42.4% were general practitioners (Table 1).

**Table 1.** Participant demographic characteristics (*n* = 1163)

	No.	%
Age range, y		
<30	170	14.6
30-39	404	34.7
40-49	318	27.3
50-59	179	15.4
≥60	92	8.0
Sex		
Male	803	69.0
Female	360	31.0
Professional background		
General practitioner	493	42.4
Urologist	211	18.1
Oncologist	266	22.9
Other	193	16.6

A total of 34.7% of participants indicated that they routinely perform prostate cancer screenings (Table 2). Notably, among participants who conduct screenings, 61.1% use PSA tests. The primary barrier to screening that participants identified was a lack of awareness about prostate cancer among patients (48.8%). To improve screening rates, participants most frequently suggested better patient education about the importance of screening (63.5%), increased training for health-care professionals (41.9%), and improved access to screening equipment (38.9%).

**Table 2.** Prostate cancer screening practices, barriers, and recommendations for improvement (*n* = 1163)

	No.	%
Routinely performing prostate cancer screening		
Yes	404	34.7
No	552	47.5
Only in certain circumstances	207	17.8
Recommended age to start prostate cancer screening, y		
<40	6	0.5
40-49	104	8.9
50-59	424	36.5
≥60	207	17.8
Other	41	3.5
Not answered	381	32.8
Methods used for prostate cancer screening^a^		
Prostate-specific antigen alone	424	36.5
Digital rectal examination alone	32	2.8
Prostate-specific antigen + digital rectal examination	282	24.2
Other	379	32.6
Main barriers to prostate cancer screening in practice^a^		
Lack of awareness about prostate cancer	596	51.2
Limited resources or equipment	346	29.8
Time constraints	246	21.2
Lack of training or knowledge	237	20.4
Prostate cancer screening is a lower priority than other health concerns	391	33.6
Other	111	9.5
Factors believed to improve the rate of prostate cancer screening^a^		
Better access to screening equipment	452	38.9
More time for patient consultations	340	29.2
Better patient education about the importance of screening	739	63.5
More training or knowledge about prostate cancer screening	487	41.9
Additional support staff (nurse practitioners, physician assistants, etc)	238	20.5
Other	62	5.3

a Multiple choices allowed.

Among the participants, 27.8% reported having fair to poor knowledge of prostate cancer screening, 65.3% had no formal training, and 88.0% expressed interest in further education on prostate cancer screening (Table 3).

**Table 3.** Knowledge and training in prostate cancer screening (*n* = 1163)

	No.	%
Proficiency in prostate cancer screening		
Excellent	210	18.1
Good	629	54.1
Fair	219	18.8
Poor	105	9.0
Formal training received on prostate cancer screening		
Yes	403	34.7
No	760	65.3
Interested in further education on prostate cancer screening		
Yes	1024	88.0
No	139	12.0

The analysis of the relationship between participants’ baseline demographic information and their preference for performing prostate cancer screening showed that as participants’ age increased, their preference for performing screening significantly increased (*P* = .001) (Table 4). In addition, female participants (*P* = .001), urologists (*P* < .001), and clinicians who received formal training (*P* < .001) were more likely to perform screening. Based on multivariable analysis, female participants (odds ratio = 4.990; *P* < .001) and urologists (odds ratio = 23.476; *P* < .001) were more likely to prefer performing PSA screening (Table 5). In addition, participants with no formal training on prostate cancer screening were less likely to perform PSA screening (odds ratio = 0.501; *P* < .001).

**Table 4.** Association between demographic factors and preferences for performing prostate cancer screening (*n* = 1163)

Variable	Yes, No. (%)	No, No. (%)	Only certain circumstances, No. (%)	*P*
Age range, y				.001
<30	51 (30.0)	99 (58.2)	20 (11.8)	
30-39	125 (30.9)	194 (48.0)	85 (21.0)	
40-49	135 (42.5)	140 (44.0)	43 (13.5)	
50-59	58 (32.4)	82(45.8)	39 (21.8)	
≥60	35 (38.0)	37 (40.2)	20 (21.7)	
Sex				.001
Male	264 (32.9)	411 (51.2)	128 (15.9)	
Female	140 (38.9)	141 (39.2)	79 (21.9)	
Professional background				<.001
General practitioner	128 (26.0)	309 (62.7)	56 (11.4)	
Urologist	134 (63.5)	31 (14.7)	46 (21.8)	
Oncologist	103 (38.7)	97 (36.5)	66 (24.8)	
Other	39 (20.2)	115 (59.6)	39 (20.2)	
Formal training received on prostate cancer screening				<.001
Yes	223 (55.3)	116 (28.8)	64 (15.9)	
No	181 (23.8)	436 (57.4)	207 (17.8)	

**Table 5.** Multivariable analysis of factors influencing the preference for performing prostate-specific antigen screening (*n* = 1163)

Covariate	Odds ratio	95% confidence interval	*P*
Age range, y			
<30	(Referent)	(Referent)	
30-39	0.780	0.539 to 1.127	.186
40-49	0.564	0.386 to 0.824	.003
50-59	0.458	0.294 to 0.715	.001
≥60	0.556	0.310 to 0.997	.049
Sex			
Male	(Referent)	(Referent)	
Female	4.990	3.597 to 6.921	<.001
Professional background			<.001
General practitioner	(Referent)	(Referent)	
Urologist	23.476	13.707 to 40.207	<.001
Oncologist	6.261	4.255 to 9.211	<.001
Other	1.280	0.874 to 1.873	.204
Formal training received on prostate cancer screening			
Yes	(Referent)	(Referent)	
No	0.501	0.376 to 0.667	<.001
Main barrier (lack of awareness about prostate cancer)			
No	(Referent)	(Referent)	
Yes	1.113	0.860 to 1.441	.415

## Discussion

In this study, we conducted a comprehensive assessment of prostate cancer screening practices among 1163 physicians across 19 countries in the Middle East and North Africa region. Our findings indicate that only 36.1% of participants perform prostate cancer screening, with the primary barrier being a lack of prostate cancer awareness among patients and the suggested solutions including better patient education and training for health-care professionals. The clinical significance of this study is its potential to inform targeted interventions that could improve prostate cancer screening rates, ultimately enhancing early detection and treatment outcomes in the Middle East and North Africa region.

The findings of our study are particularly important given that a significant number of patients with prostate cancer in the Middle East and North Africa region present with metastatic disease at the time of diagnosis. This fact is evidenced by a retrospective analysis of 1136 patients with prostate cancer across 6 Middle East and North Africa countries, which revealed that 54% of the patients had metastatic disease at the time of initial presentation in 2021.^7^ Similarly, another study of 615 patients with prostate cancer from 4 Middle East and North Africa countries between 2012 and 2019 showed that 42% of patients presented with metastatic disease at the time of diagnosis.^24^ In contrast, less than 7% of patients with prostate cancer in the United States are diagnosed with metastatic disease at the time of initial presentation.^25-28^

Several points require further discussion. First, our study revealed that the primary barrier to prostate cancer screening is a lack of awareness among patients. This finding is particularly important because major guidelines emphasize the shared decision-making approach to screening, which relies heavily on patient awareness and understanding.^18-21^ Recent studies have also highlighted low prostate cancer awareness in the Middle East and North Africa region, noting common misconceptions and low awareness of PSA screening.^10^ It is also important to note that the second-most common barrier to screening was the perception that prostate cancer screening is a lower priority than other health concerns. Although cancer screening is essential, health-care professionals often prioritize immediate and high-burden diseases because of resource constraints, patient preferences, and knowledge gaps.^29^^,^^30^ This fact was also reflected in our study, with 29.8% of participants reporting limited resources and 20.4% citing lack of training among the main barriers to prostate cancer screening.

Second, our study identified better patient education as the most critical factor to improve the rate of prostate cancer screening, with 63.5% of participants highlighting it as a top priority. Studies highlight that patient education interventions, particularly those tailored to the specific needs and cultural context of the target population, can effectively address barriers to cancer screening and improve participation rates.^31^^,^^32^ Strategies such as one-on-one counseling,^33^^,^^34^ visual aids,^35^^,^^36^ reminders,^37^ and using community health workers trained in culturally sensitive approaches^38^ have shown promising results in increasing cancer screening uptake. It is also important to note that 65.3% of the participants in our study reported having no formal training on prostate cancer screening, and 41.9% indicated that more training would improve the rate of screening. This finding aligns with multiple studies that emphasize the positive impact of targeted training and education programs for health-care professionals on improving their knowledge and, ultimately, cancer screening rates among their patients.^39-41^ Improving both patient and health-care professional knowledge on prostate cancer screening is particularly important because shared decision making relies heavily on informed discussions about the benefits and risks of screening.

Finally, although our study provides valuable insights into the status of prostate cancer screening in the Middle East and North Africa region, it has several limitations that should be acknowledged. First, the recruitment method, which relied on the discretion of local team leaders across 19 countries, may have introduced selection bias. Although the majority of participants were general practitioners, urologists, or oncologists, 16.6% of the participants indicated their specialty as “Other,” which could affect the generalizability of the findings. In addition, the intended sample size for ensuring statistical significance may not have been met in each location, as reflected by the variation in participant numbers, which ranged from 22 to 84 per location. The nonresponse rate was 3.2%, and specific data on the reasons for nonparticipation were not collected, which could further affect the generalizability of the results. Finally, because of the study's design and collaborative agreements, the study aimed to emphasize the regional perspective on prostate cancer screening. Although this method supports a comprehensive analysis beneficial to regional health initiatives, it limits the examination of variations at the individual country level. To address this limitation, future research should focus on country-specific analyses to provide insights for tailored interventions that account for each country’s unique health-care systems and economic conditions.

This study provides valuable insights into the current status of prostate cancer screening in the Middle East and North Africa region, highlighting significant barriers, such as a lack of patient awareness and formal training among health-care professionals. Given the high prevalence of advanced-stage prostate cancer at diagnosis in this region, there is an urgent need for early detection initiatives. To enhance screening rates, it is essential to implement tailored patient education, culturally sensitive outreach programs, and comprehensive training for health-care professionals. Future research should focus on country-specific analyses to develop effective, localized interventions that would improve patient outcomes and reduce the burden of prostate cancer across the Middle East and North Africa region.

## Supplementary Material

pkaf019_Supplementary_Data

## Data Availability

The data underlying this article are available in the article and in its online supplementary material.
