# Supplementary material for: Prostate cancer screening in the Middle East and North Africa: a cross-sectional study on current practices
Source: JNCI Cancer Spectr. 2025 Feb 8;9(2):pkaf019. doi: 10.1093/jncics/pkaf019 (PMC11927531; doi:10.1093/jncics/pkaf019)
Supplement: pkaf019_Supplementary_Data [file pkaf019_supplementary_data.zip › Supplementary Material.docx]

**Supplementary Material**

**Data collection tool: Prostate Cancer Screening Questionnaire**

1. **Demographic information**

- Please specify your age range:

Under 30

30-39

40-49

50-59

60 and above

- Please specify your gender:

Male

Female

- Please specify your professional background:

General practitioner

Urologist

Oncologist

Other (please specify):

1. **Screening Practice**

- Do you routinely perform prostate cancer screening on your patients?

Yes

No

Only in certain circumstances (please specify):

- What is the typical age at which you start screening your patients for prostate cancer?

Under 40

40-49

50-59

60 and above

Other (please specify):

- What methods do you typically use for prostate cancer screening? (Choose all that apply)

Prostate-specific antigen (PSA) test

Digital rectal exam (DRE)

Other (please specify):

1. **Barriers to Screening**

- What are the main barriers to prostate cancer screening in your practice? (Choose all that apply)

Lack of awareness about prostate cancer

Limited resources or equipment

Time constraints

Lack of training or knowledge

Prostate cancer screening is lower priority compared to other health concerns

Other (please specify):

- What factors do you believe would improve the rate of prostate cancer screening in your practice? (Choose all that apply)

Better access to screening equipment

More time for patient consultations

Better patient education about the importance of screening

More training or knowledge about prostate cancer screening

Additional support staff (nurse practitioners, physician assistants, etc.)

Other (please specify):

1. **Awareness and Education**

- How would you rate your own knowledge about prostate cancer and its screening methods?

Excellent

Good

Fair

Poor

- Have you received any formal training on prostate cancer screening?

Yes

No

- Would you be interested in further education or training on prostate cancer screening?

Yes

No
